# γδT cells, a key subset of T cell for cancer immunotherapy

**DOI:** 10.3389/fimmu.2025.1562188

**Published:** 2025-03-28

**Authors:** Jianzhen Lv, Zheng Liu, Xiangting Ren, Siyuan Song, Yan Zhang, Yi Wang

**Affiliations:** ^1^ Guangxi Key Laboratory of Efficacy Study on Chinese Materia Medica, Institute of Traditional Chinese and Zhuang-Yao Ethnic Medicine, Guangxi University of Chinese Medicine, Nanning, Guangxi, China; ^2^ Pathology Department, University of Texas MD Anderson Cancer Center, Houston, TX, United States; ^3^ Medical School, Guangxi University of Chinese Medicine, Nanning, Guangxi, China; ^4^ Department of Neuroscience, Baylor College of Medicine, Houston, TX, United States; ^5^ Department of Geriatrics, Sichuan Provincial People’s Hospital, University of Electronic Science and Technology of China, Chengdu, China; ^6^ Department of Critical Care Medicine, Sichuan Provincial People’s Hospital, University of Electronic Science and Technology of China, Chengdu, Sichuan, China; ^7^ Clinical Immunology Translational Medicine Key Laboratory of Sichuan Province, Center of Organ Transplantation, Sichuan Academy of Medical Science and Sichuan Provincial People’s Hospital, Chengdu, Sichuan, China

**Keywords:** γδT cells, tumor immunotherapy, phosphoantigens, chimeric antigen receptor (CAR), cytokine production, stress-induced ligands, adoptive cell therapy, cancer treatment

## Abstract

γδT cells represent a unique and versatile subset of T cells characterized by the expression of T-cell receptors (TCRs) composed of γ and δ chains. Unlike conventional αβT cells, γδT cells do not require major histocompatibility complex (MHC)-dependent antigen presentation for activation, enabling them to recognize and respond to a wide array of antigens, including phosphoantigens, stress-induced ligands, and tumor-associated antigens. While γδT cells are relatively rare in peripheral blood, they are enriched in peripheral tissues such as the skin, intestine, and lung. These cells play a crucial role in tumor immunotherapy by exerting direct cytotoxicity through the production of inflammatory cytokines (e.g., interferon-gamma (IFN-γ), tumor necrosis factor-alpha (TNF-α), and interleukin-17 (IL-17)) and cytotoxic molecules (e.g., perforin and granzyme). Recent advances in γδT cell research have elucidated their mechanisms of tumor recognition, including the detection of phosphoantigens and stress-induced ligands like MICA (MHC class I polypeptide-related sequence A), MICB (MHC class I polypeptide-related sequence B), and ULBP (UL16-binding protein). Furthermore, various strategies to enhance γδT cell-based tumor immunotherapy have been developed, such as *in vitro* expansion using phosphoantigen-based therapies, cytokine stimulation, and chimeric antigen receptor (CAR)-γδT cell engineering. These advancements have shown promising results in both preclinical and clinical settings, paving the way for γδT cells to become a powerful tool in cancer immunotherapy. This review highlights the key mechanisms, functions, and strategies to harness the potential of γδT cells for effective tumor immunotherapy.

## 1 Introduction

A unique subset of T cells, γδT cells, are characterized by expressing a T-cell receptor (TCR) composed of γ and δ chains, instead of the α and β chains of conventional αβT cells (1). For the activation of γδT cells, they do not need major histocompatibility complex (MHC)-dependent antigen presentation (2). This unique characterization allows γδT cells to recognize and respond to a wide range of antigens without antigen presentation cells (APC). However, γδT cells are relatively rare in the circulation of peripheral blood. The majority of γδT cells are resident in peripheral tissues, including the skin, intestine, and lung (3). As a promising immune cell subset in tumor immunotherapy, γδT cells possess innate-like properties, including the recognition of a variety of tumor-associated antigens without the help of APC (4). Further, γδT cells are cytotoxic by producing inflammatory cytokine attacking the tumor cells (5). The target of γδT cells includes a broad range of solid tumors and hematological malignancies by exploiting stress signals on tumor cells, tumor-associated antigens, and altered metabolic products such as phosphoantigens (pAgs) (6). Therefore, we updated the recent research on the γδT cells, focusing on its tumor recognition to adoptive transfer potential for tumor immunotherapy.

## 2 Mechanisms of tumor recognition by γδT cells and cytokine production

γδT cells could recognize non-peptide antigens such as pAgs, especially recognized by a subset of γδT cells named Vγ9Vδ2 T cells (7). It could also recognize stress-induced ligands, such as MICA (MHC class I polypeptide-related sequence A), MICB (MHC class I polypeptide-related sequence B), ULBP (UL16-binding protein), and heat shock proteins (HSPs), without the need for MHC.

### 2.1 Phosphoantigens recognition by Vγ9Vδ2 T cells

In human peripheral blood, the most abundant subset of γδT cells are Vγ9Vδ2 T cells. This subset of cells is activated by pAgs, including isopentenyl pyrophosphate (IPP) or dimethylallyl pyrophosphate (DMAPP) (8–10). These pAgs have been documented to be generated during microbial infections or tumorigenesis. Through the recognition of these pAgs by the TCR (T-cell receptor) of Vγ9Vδ2, γδT cells are activated thereby producing cytokines and immune attack on tumor cells (11).

Activated Vγ9Vδ2 γδT cells can produce and secrete pro-inflammatory cytokines, including interferon-γ (IFN-γ) (12), tumor necrosis factor-α (TNF-α) (13), interleukin-17 (IL-17) (14), and interleukin-22 (IL-22) (15); immune-regulatory cytokines, such as interleukin-10 (IL-10) (16, 17); cytotoxic molecules, including granzyme and perforin (18); and other cytokines, such as granulocyte-macrophage colony-stimulating factor (GM-CSF), interleukin-4 (IL-4), and interleukin-5 (IL-5) (16, 19).

**Figure 1:**
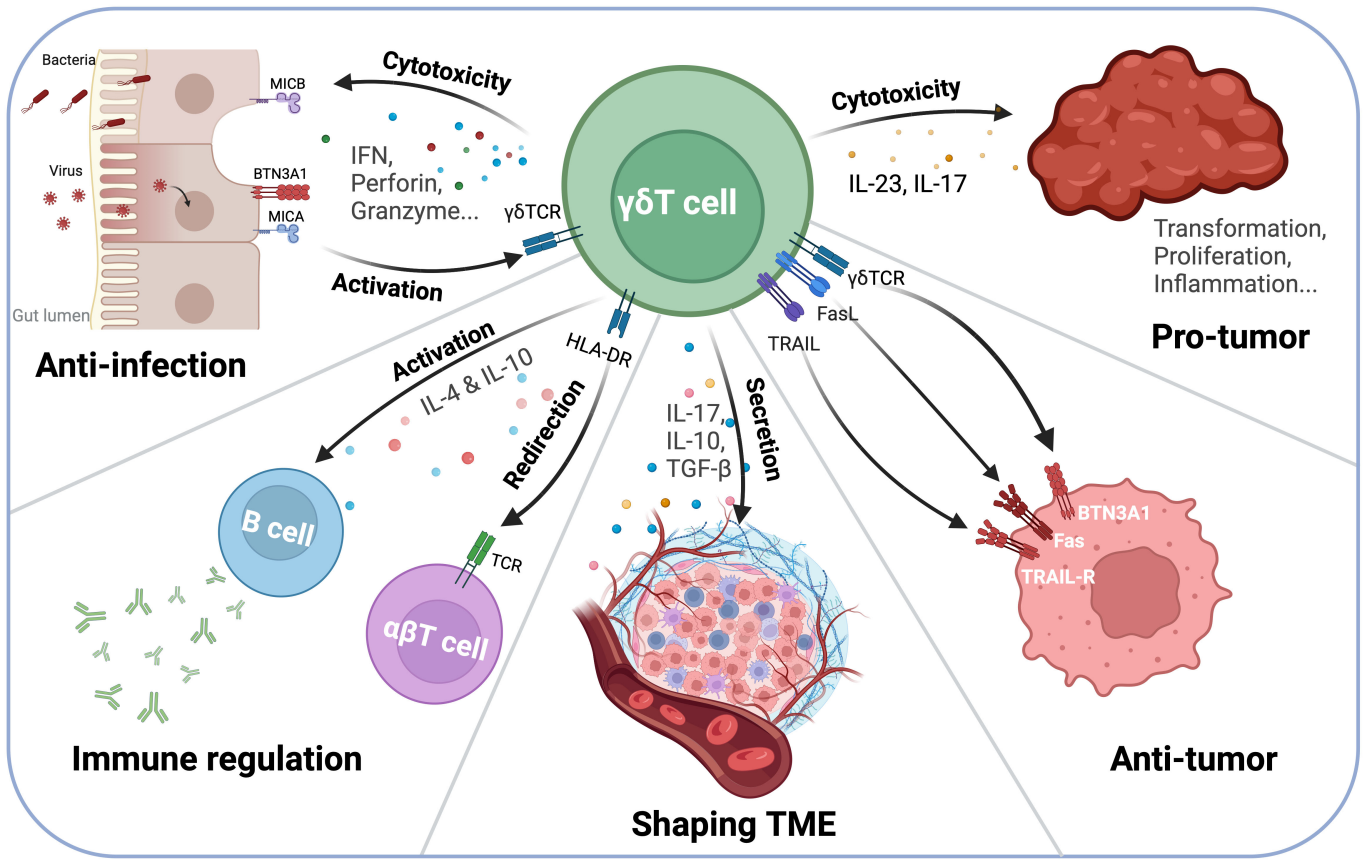
γδT Cell interactions with the immune system and Tumor Microenvironment (TME). γδT cells play a dual role in tumor immunity, exhibiting both anti-tumor and pro-tumor functions depending on the microenvironment. On one hand, γδT cells secrete IFN-γ, perforin, and granzyme, mediating direct tumor cell lysis. Additionally, they interact with antigen-presenting cells (APCs) and B cells via HLA-DR, influencing B cell class switching and antibody production. On the other hand, γδT cells can contribute to a pro-tumor environment through IL-17 and IL-23 secretion, which promotes inflammation and tumor progression. Their ability to shape the TME highlights their potential as both therapeutic targets and immunotherapy agents.

Among these, IFN-γ is a major cytokine that plays a crucial role in modulating the immune response by activating macrophages, enhancing antigen presentation, and promoting the differentiation of Th1 cells for antitumor function (12). TNF-α is another important pro-inflammatory cytokine produced by γδT cells (13). It could induce tumor cell apoptosis via immune activation. TNF-α can also contribute to the elimination of infected or cancerous cells. IL-17 is produced by a subset of γδT cells, particularly Vγ9Vδ2 cells (14). IL-17 promotes inflammation and can recruit neutrophils and other immune cells to sites of infection or tumor growth. However, its role in the context of cancer can be more complex, as it can have both tumor-promoting and tumor-suppressing effects depending on the tumor microenvironment (TME). IL-22 is another cytokine that can be produced by activated γδT cells, particularly in mucosal immunity (15). It plays a key role in tissue repair and protection from infection, but it can also have a dual role in promoting tumor growth in certain contexts. GM-CSF is also produced by γδT cells and can promote the differentiation and activation of macrophages and dendritic cells, thereby enhancing the overall immune response and antigen presentation (19). Under certain conditions, γδT cells can produce IL-4 and IL-5, which are typically associated with the regulation of humoral immunity and eosinophil activation (16). IL-10 is an anti-inflammatory cytokine that can be produced by γδT cells during the regulation of immune responses or inflammation (20). However, its production is generally lower compared to other cytokines, such as IFN-γ or TNF-α. Granzyme and perforin are key cytotoxic molecules produced by activated γδT cells that contribute to the direct killing of infected or cancerous cells. The release of these molecules is an essential mechanism of γδT cell-mediated cytotoxicity (18) (**Figure 1**).

Meanwhile, tumor cells can overexpress mevalonate pathway metabolites, including mevalonate, isopentenyl pyrophosphate (IPP) (21), dimethylallyl pyrophosphate (DMAPP) (22), geranyl pyrophosphate (GPP), farnesyl pyrophosphate (FPP), geranylgeranyl pyrophosphate (GGPP), cholesterol, coenzyme Q, dolichol, prenylated proteins, steroid hormones (23, 24). These mevalonate pathway metabolites play a crucial role in the function of γδT cells (25). IPP is a potent stimulatory antigen for γδT cells, particularly the Vγ9Vδ2 subset (26). However, the presentation of small pAg, including IPP and DMAPP, is dependent on butyrophilin (BTN) family members, specifically BTN3A1 and BTN2A1 (27). BTN3A1, also known as CD277, is an intracellular sensor that binds to pAgs, while BTN2A1 facilitates their recognition by γδTCR (28, 29). Together, BTN3A1 and BTN2A1 form a molecular complex that enables γδT cell activation upon pAg accumulation.

In normal cells, IPP concentrations are low, but in cancer cells, the mevalonate pathway is often upregulated, leading to IPP accumulation (21). BTN3A1-mediated sensing of IPP leads to a conformational change that allows BTN2A1 to interact with the Vγ9Vδ2 TCR, triggering γδT cell proliferation and activation (30). This activation results in tumor cell recognition and cytotoxic response γδT cells a promising candidate for cancer immunotherapy (31). Further, IPP secreted from zoledronic acid (ZOL)-stimulated myeloma cells can activate the chemotaxis of γδT cells (32). ZOL, a nitrogen-containing bisphosphonate, inhibits farnesyl pyrophosphate synthase in the mevalonate pathway, resulting in IPP accumulation, which enhanced γδT cells recruitment and activation (33). Besides, the activation of γδT cells by IPP can lead to the secretion of pro-inflammatory cytokines such as TNF-α, which can further increase the immune responses (34). Additionally, the mevalonate pathway metabolites can influence the differentiation and effector functions of γδT cells, contributing to their role in immune surveillance and anti-tumor immunity (35). Therefore, the application potential of nitrogen-containing bisphosphonates as ZOL to expand γδT cells *ex vivo* has been successful in various cancer types (36, 37). These expanded cells can then be used in adoptive cell transfer therapy.

### 2.2 Stress-induced ligands and heat shock proteins

MICA, MICB, and ULBP are members of the NKG2D (Natural Killer Group 2, Member D) ligand family, which play a crucial role in the immune response, particularly in the activation of natural killer (NK) cells and γδT cells (38, 39). MICA, MICB, and ULBP are typically expressed at low levels in healthy cells (40). However, their expression is significantly upregulated under conditions of cellular stress, such as infection, DNA damage, or transformation in cancer cells (41, 42). This upregulation recruits the NK cells and γδT cells to these abnormal cells under stress. During tumor immunosurveillance, the expression of MICA, MICB, and ULBP on tumor cells can be recognized by NKG2D, a NK-like activating receptor (NKR) expressed on γδT cells, particularly in Vγ9Vδ2 subset (43). Subsequently, NKG2D binds to these ligands to form NKG2D–NKG2D ligand (NKG2D–NKG2DL) axis, leading to the activation of γδT cells, triggering cytotoxicity and cytokine production (IFN-γ, TNF-α, etc) (44).

HSPs are a family of highly conserved and immunogenic proteins that are expressed in response to various stress conditions. In γδT cells, HSP60 helps maintain the integrity and function of cellular proteins, especially under stress conditions. HSP60 can also act as an immunomodulatory molecule. It can be recognized by γδT cells and other immune cells, leading to the activation of immune responses (45). Also, in γδT cells, HSP70 can enhance cell survival and function during stress. HSP70 can bind to antigens and present them to immune cells, including γδT cells, thereby activating the immune response (46, 47). HSP72 by LPS-stimulated neutrophils facilitates γδT cell-mediated killing (48). HSP90 is overexpressed in cancer cells, contributing to survival and growth. Therefore, HSP90 plays a role in the activation of γδT cells by targeting this molecule (49). Overall, these heat shock proteins can be recognized via the γδT cells and NKG2D, further contributing to cytotoxicity and immune response against cancer cells.

## 3 γδT cells in tumor immunotherapy

Upon activation, γδT cells exhibit three major functions for tumor immunotherapy, which are cytotoxicity, cytokine production, tissue-resident and memory responses.

### 3.1 Cytotoxicity

#### 3.1.1 Mechanisms

**Figure 2:**
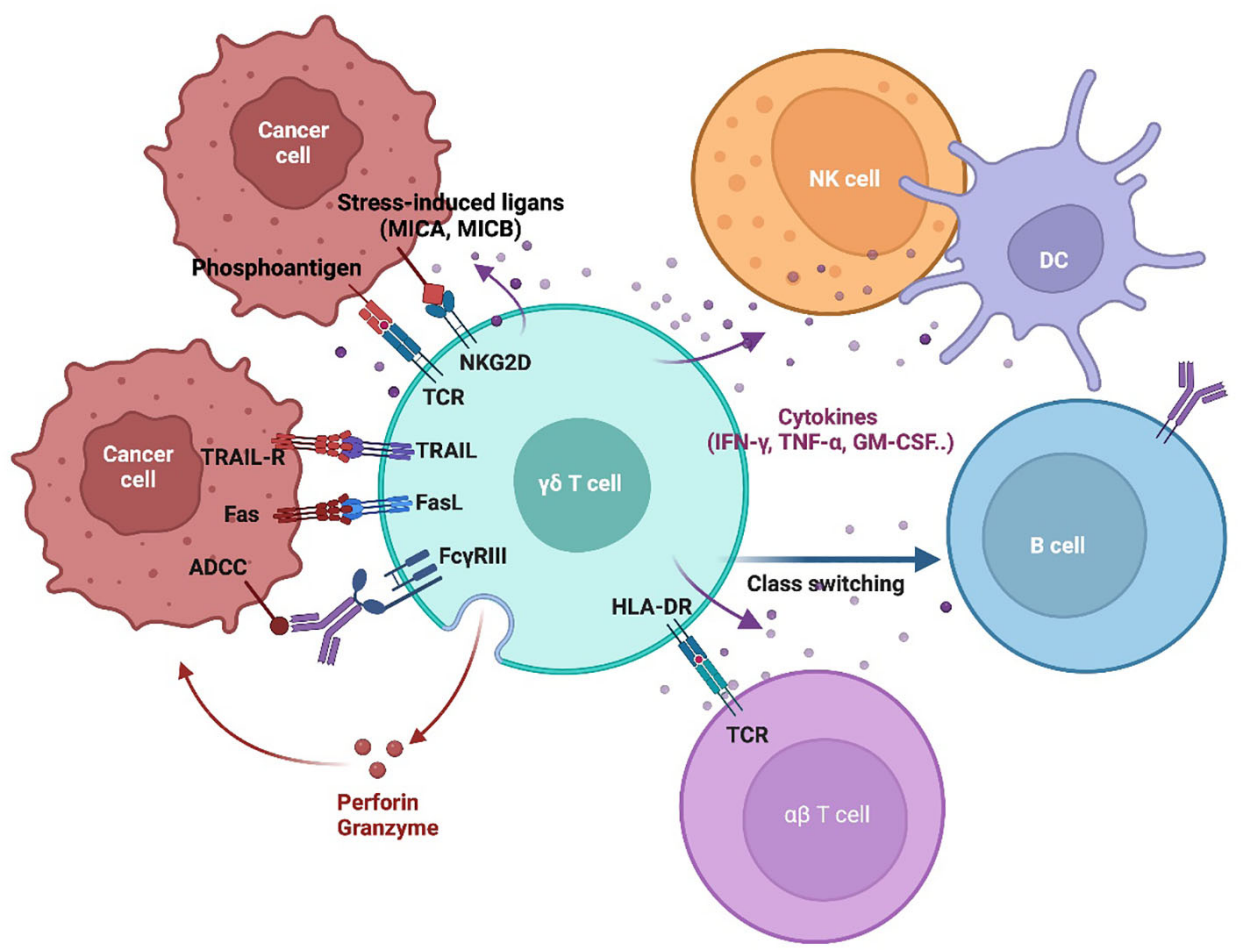
γδT Cell tumor recognition and cytotoxic mechanisms. This schematic illustrates the key mechanisms by which γδT cells recognize and eliminate tumor cells. γδT cell activation is initiated through TCR engagement with phosphoantigens and stress-induced ligands (MICA/MICB) on tumor cells. Additionally, γδT cells express NK-like activating receptors (NKRs), such as NKG2D, which enhance recognition and activation. γδT cell-mediated cytotoxicity through multiple mechanisms, including the TRAIL/TRAIL-R and Fas/FasL pathways, which induce the tumor cell apoptosis. They also release perforin and granzyme, leading to direct cytolysis. Engagement of NKG2D with tumor-expressed ligands further enhances tumor cell destruction. Moreover, γδT cell participate in antibody-dependent cellular cytotoxicity (ADCC) via FcγRIII, enabling them to target opsonized tumor cells. Beyond direct cytotoxicity, γδT cells interact with other immune cells, including NK cells, dendritic cells (DCs), B cells, and αβ T cells, playing a role in immune modulation and influencing the broader anti-tumor response.

Activated γδT cells eliminate tumor cells through multiple mechanisms (**Figure 2**). They target and eliminate tumor cells independently of MHC restriction, making them particularly advantageous for immunotherapy applications.

##### 3.1.1.1 Granule exocytosis pathway

One of the primary mechanisms by which γδT cells eliminate tumor cells is through granule exocytosis, a process that relies on the release of cytotoxic molecules. Upon activation, γδT cells degranulate and release perforin, which forms pores in tumor cell membrane, allowing granzyme B to enter the target cell (50). Once inside, granzyme B activates caspase-dependent apoptotic pathways, leading to tumor cell death. This mechanism is rapid and highly effective, making it a crucial cytotoxic pathway utilized by γδT cells in anti-tumor immunity.

##### 3.1.1.2 Death receptor-mediated apoptosis

γδT cells also induce apoptosis through death receptor pathways, including Fas-FasL, TRAIL (TNF-related apoptosis-inducing ligand), and TNF-α-mediated signaling. Fas ligand (FasL) expressed on activated γδT cells can bind to Fas (CD95) on tumor cells, triggering caspase-dependent apoptosis of the target cells (51). Additionally, γδT cells also produce TNF-α, which binds to TNF receptors on tumor cells, further promoting apoptosis via extrinsic signaling cascades (52). Moreover, γδT cells can engage in TRAIL signaling, where TRAIL binds to TRAIL receptors (DR4/DR5) on tumor cells, initiating apoptosis. Preclinical studies in lung and breast cancer models have shown that TRAIL-dependent cytotoxicity effectively eliminates tumor cells (53).

##### 3.1.1.3 NK-like activating receptors-mediated cytotoxicity

In addition to their TCR-mediated responses, γδT cells express NKRs, which enhance their ability to recognize and eliminate tumor cells (54). One of the most well-characterized NKRs expressed on γδT cells is NKG2D, which binds to MICA, MICB, and ULBPs, stress-induced ligands commonly upregulated in tumor cells (55). This interaction enhances γδT cell cytotoxicity by promoting activation and degranulation. Additionally, γδT cells express DNAM-1 (CD226), which binds to CD155 (PVR) and CD112 (Nectin-2), further strengthening tumor recognition and immune activation (56).

γδT cells also express natural cytotoxicity receptors (NCRs), including NKp30, NKp44, and NKp46, which interact with ligands such as B7-H6, PCNA, and Heparan sulfate (57, 58). These receptors, typically associated with NK cells, contribute to the ability of γδT cells to recognize and eliminate tumor cells that evade classical T cell surveillance (59). The presence of NKRs on γδT cells highlights their hybrid functional profile, bridging innate and adaptive immune responses for effective tumor control (60).

##### 3.1.1.4 Antibody-dependent cellular cytotoxicity

Beyond direct killing, γδT cells mediate ADCC, an essential mechanism for monoclonal antibody (mAb)-based cancer therapies (61). γδT cells express Fc receptors (FcγRIII/CD16), allowing them to recognize and kill tumor cells opsonized by therapeutic antibodies (62). Clinical evidence suggests that γδT cells enhance the efficacy of mAb therapies, such as rituximab (anti-CD20) in B-cell malignancies and trastuzumab (anti-HER2) in breast cancer (63, 64). A Phase I/II trial evaluating the combination of ex vivo-expanded γδT cells with trastuzumab demonstrated enhanced tumor regression in HER2^+^ breast cancer patients, indicating the clinical potential of ADCC-mediated γδT cell therapy (65).

#### 3.1.2 Preclinical and clinical findings

γδT cells have been tested across various preclinical and clinical models, demonstrating potent tumor cytotoxicity in both solid and hematologic malignancies. Preclinical studies in ovarian and lung cancer have shown that adoptively transferred γδT cells efficiently infiltrate tumors, secrete IFN-γ, and kill tumor cells, leading to significant tumor growth inhibition (66). Clinically, several trials have demonstrated the feasibility of γδT cell-based therapies. A Phase I clinical trial (NCT03183232) demonstrated the safety and feasibility of autologous γδ T cell therapy in patients with advanced hepatocellular carcinoma (HCC), showing prolonged disease stabilization. Another clinic trial in glioblastoma showed that γδT cells, when combined with low-dose chemotherapy, led to tumor shrinkage and prolonged survival in patients with recurrent disease (67). In multiple myeloma, γδT cells expanded with ZOL and IL-2 have been investigated, demonstrating safe administration and tumor regression in patients resistant to conventional therapies (6).

##### 3.1.2.1 Cytokine production

The cytokines generated and released by γδT cells have been addressed in detail in the previous section. IFN-γ, TNF-α, and IL-17 produced by γδT cells could enhance the immune response, promote tumor inflammation, and recruit other immune cells (68). IFN-γ can have direct anti-tumor effects by upregulating MHC class I molecules on tumor cells, enhancing their recognition by other immune cells like cytotoxic T cells (CD8^+^ T cells) and NK cells.

In glioblastoma, a highly aggressive brain tumor, γδT cells have been shown to secrete IFN-γ, which sensitizes tumor cells to immune checkpoint blockade therapy. Additionally, in colorectal cancer, γδ T cell-derived IL-17 has been implicated in shaping the tumor microenvironment by recruiting neutrophils and enhancing immune responses.

##### 3.1.2.2 Tissue-resident and memory responses

As the majority of γδT cells are resident in tissues, they serve as tissue-resident lymphocytes, providing long-term surveillance and response to tumor recurrence. The migration and tissue colonization of γδT cells in specific tissues, such as the small intestine, are regulated by chemotactic signals, adhesion molecules, and signaling pathways, including CCR9/CCL25 pathway (69). Meanwhile, different subsets of γδT cells express distinct chemokine receptors that determine their homing properties. The Vδ2 subset expresses CCR5 and CXCR3 (70, 71), which are associated with Th1 cell functions, while the Vδ1 subset expresses CXCR1 and CCR2 (70).

For the adaptive immunity, γδT cells also exhibit memory-like properties, similar to conventional αβT cells. They can undergo clonal expansion and differentiation upon antigen encounter, leading to the formation of memory cells. Therefore, CAR-γδT cells have been designed to enhance the cytotoxicity of γδT cells against lymphoid malignancies (72, 73). For example, a preclinical study demonstrated that CAR-γδT cells engineered to target CD19 effectively eliminated B-cell acute lymphoblastic leukemia (B-ALL). Another recent study reported that γδ T cell-based immunotherapy enhanced responses to standard chemotherapy in patients with ovarian cancer, highlighting their potential in solid tumor treatment.

## 4 Challenges and strategies for enhancing γδT cell-based tumor immunotherapy

Despite the promising therapeutic potential of γδT cells in cancer immunotherapy, several challenges remain in their clinical application. These challenges include optimizing ex vivo expansion protocols, enhancing persistence after infusion, addressing functional heterogeneity, and overcoming immunosuppressive tumor microenvironments (TME). Addressing these limitations is crucial for improving the efficacy and durability of γδT cell-based therapies.

### 4.1 Challenges and limitations in γδT cell-based therapies

#### 4.1.1 Expansion protocols and manufacturing challenges

A major challenge in γδT cell-based immunotherapy is the variability and inefficiency of ex vivo expansion protocols, which are critical for generating sufficient cell numbers for clinical applications (74). The expansion of γδT cells, particularly the Vγ9Vδ2 subset, relies on pAg-based activation using compounds such as zoledronate or synthetic pAgs (e.g., IPP, DMAPP) (75). While effective, this approach suffers from inconsistencies across different culture conditions and donor-dependent variability, leading to difficulties in standardization and scalability. Furthermore, prolonged ex vivo expansion can lead to functional exhaustion, reducing the cytotoxic potential of γδT cells before infusion (76). In contrast, Vδ1 γδT cells, which have shown greater efficacy against solid tumors, are more challenging to expand using conventional methods (77). Current protocols for expanding Vδ1 cells are inefficient and often yield a heterogeneous population, making it difficult to achieve reproducible therapeutic effects. Moreover, the transition from research-grade expansion to Good Manufacturing Practice (GMP)-compliant protocols poses an additional hurdle, requiring refined methods that ensure both clinical efficacy and regulatory approval.

#### 4.1.2 Limited *in vivo* persistence after infusion

Unlike αβT cells, γδT cells exhibit limited proliferation and persistence *in vivo* following adoptive transfer, which significantly restricts their long-term anti-tumor effects (4). One of the primary reasons for this limitation is insufficient cytokine support in the host environment, which fails to sustain γδT cell survival and function. In particular, γδT cells rely on cytokines such as IL-2, IL-15, and IL-21 for survival, but their availability *in vivo* is often inadequate for robust expansion post-infusion (78). Additionally, poor metabolic fitness and an inability to efficiently utilize energy sources in the tumor microenvironment further hinder γδT cell longevity. Another major concern is the emergence of exhaustion markers such as PD-1, TIM-3, and LAG-3 on γδT cells after repeated stimulation, leading to a progressive decline in their cytotoxic activity (79). This exhaustion phenotype is exacerbated in solid tumors, where γδT cells encounter persistent antigenic stimulation and an immunosuppressive milieu that dampens their efficacy (79). Furthermore, suboptimal engraftment in the tumor microenvironment, due to poor homing signals and competition with other immune cells, limits the ability of γδT cells to accumulate and exert sustained anti-tumor effects (66). Overcoming these barriers is crucial for improving the therapeutic durability of γδT cell-based therapies.

#### 4.1.3 Functional heterogeneity of γδT cells

γδT cells represent a highly heterogeneous immune population, with different subsets exhibiting distinct functional properties and tumor-targeting capabilities. The Vγ9Vδ2 subset, which is predominantly found in peripheral blood, has shown robust cytotoxic activity, particularly in hematologic malignancies, where these cells efficiently target tumor cells. In contrast, Vδ1 γδT cells, which are enriched in epithelial tissues, play a more prominent role in targeting solid tumors due to their tissue-resident nature and ability to interact with the TME. However, this variability complicates the application of γδT cell-based therapies, as the most effective subset for tumor elimination depends not only on the tumor type but also on the specific TME characteristics, such as cytokine milieu and immune cell composition (77).

In hematologic cancers, where γδT cells have better accessibility and can easily encounter tumor cells circulating in the bloodstream or bone marrow, Vγ9Vδ2 cells are particularly effective (80). However, in solid tumors, the limited infiltration of γδT cells into the dense tumor stroma, compounded by the immunosuppressive TME, restricts their functionality. Furthermore, while many γδT cells exert potent anti-tumor effects through the production of pro-inflammatory cytokines like IFN-γ and TNF-α, certain subsets, including IL-10-producing γδT cells, have been identified as immunosuppressive, particularly in solid tumors. These IL-10-producing γδT cells contribute to immune suppression and tumor immune evasion, raising concerns that γδT cell therapies, if not properly controlled, could inadvertently promote tumor progression (81). This highlights the critical need to develop strategies that selectively expand the most cytotoxic γδT cell subsets (such as those producing IFN-γ) and minimize the expansion of regulatory subsets that may hinder therapeutic efficacy, especially in solid tumor settings. Moreover, the differentiation and plasticity of γδT cells are influenced by factors such as cytokine exposure and metabolic signals, which further contribute to their heterogeneity across different tumor types.

#### 4.1.4 Immunosuppression of the TME

The immunosuppressive nature of the TME represents a significant barrier to the effectiveness of γδT cell-based immunotherapies, with solid tumors posing a particularly challenging environment for γδT cell infiltration and function. Tumors evade γδT cell-mediated cytotoxicity through several mechanisms, including the downregulation of NKG2D ligands (e.g., MICA, MICB, ULBPs), which are crucial for γδT cells to efficiently recognize and attack tumor cells (82). In solid tumors, the lack of these activating ligands prevents γδT cells from initiating robust anti-tumor responses, allowing tumors to escape immune surveillance (83). Additionally, solid tumors often secrete immunosuppressive factors, such as TGF-β, IL-10, and adenosine, which inhibit γδT cell activation, proliferation, and function (84). TGF-β, in particular, has been shown to drive the conversion of cytotoxic γδT cells into regulatory subsets, further compromising the effectiveness of γδT cell therapy, particularly in solid tumor contexts (85).

While hematologic tumors are more readily accessible to γδT cells in circulation, where they do not face the same physical barriers as solid tumors, they still present challenges (86). Tumor cells in the bloodstream can evade recognition by γδT cells through mechanisms such as immune checkpoint molecule expression (e.g., PD-L1) or cytokine-driven suppression (87). In solid tumors, however, the physical barriers, including the dense extracellular matrix and abnormal vasculature, impede immune cell trafficking, and hypoxic conditions can further reduce γδT cell efficacy (88). Once γδT cells infiltrate the tumor, chronic exposure to inhibitory signals such as PD-1/PD-L1 interactions induces T cell exhaustion, which limits their functional capacity and persistence. The challenge in solid tumors, therefore, lies not only in the tumor’s ability to block γδT cell activity but also in the difficulties γδT cells face in infiltrating and surviving in these environments. Strategies to overcome these suppressive mechanisms are essential for improving the infiltration, activation, and persistence of γδT cells in the TME (53). These strategies include combining γδT cell therapy with immune checkpoint inhibitors (e.g., anti-PD-1, anti-CTLA-4), engineering γδT cells to resist TME-induced exhaustion, and utilizing approaches such as local tumor irradiation or metabolic reprogramming to enhance γδT cell recognition and infiltration in solid tumor settings.

### 4.2 Optimization of γδT cell expansion and activation

Efficient expansion and activation of γδT cells are critical for their clinical application, yet several challenges still remain, which necessitate refined strategies to improve γδT cell proliferation, maintain functionality, and standardize large-scale production.

#### 4.2.1 pAgs based activation

ZOL and synthetic pAgs-based therapies are one of the most prominent ones for *in vitro* expansion and activation of γδT cells (89). Synthetic or modified pAgs, such as IPP or DMAPP analogs can be used to activate Vγ9Vδ2 T cells in patients (22). Bromohydrin pyrophosphate (BrHPP), ZOL, and 2-methyl-3-butenyl-1-pyrophosphate (2M3B1PP) are used to increase the concentration of pAgs in tumor cells, directly activating and expanding γδT cells, and thereby promoting their cytotoxicity (89–91). High concentrations of ZOL (100 μM) stimulation at a short period could induce Vδ2T cell expansion. Besides, zoledronic acid and other bisphosphonates can upregulate pAgs levels in the mevalonate pathway, indirectly activating Vγ9Vδ2 T cells (92).

#### 4.2.2 Cytokine-based expansion

The expansion of γδT cells can be greatly increased by specific cytokines, i.e., IL-15, IL-12, IL-18, and IL-23. IL-15 is critical for the activation, survival, and expansion of γδT cells. IL-15 has been investigated in clinical trials as an adjuvant therapy to enhance γδT cell function and boost their numbers *in vivo* (11). IL-12 (8, 12, 33) and IL-18 (11) could promote the differentiation and activation of γδT cells into effector cells that produce IFN-γ and TNF-α, enhancing their anti-tumor activity. IL-23 could promote IL-17 production by γδT cells, which can contribute to tumor rejection and immune modulation (14, 15). Meanwhile, the addition of vitamin C and its more stable derivative, L-ascorbic acid 2-phosphate (pVC), could also significantly increase the proliferation and cytotoxic activity of Vδ2T cells (93).

#### 4.2.3 Artificial APCs

Along with the cytokines and pAgs, the application of artificial APCs to express costimulatory molecules and antigens can also improve the expansion and activation of γδT cells. NKG2D-NKG2DL axis plays a crucial role in γδT cell activation and expansion (94). Therefore, KG2D agonists could enhance NKG2D signaling and increase the activation and cytotoxicity of NKG2D^+^ γδT cells. CD20-specific immune-ligands engaging NKG2D could improve the cytotoxicity of γδT cells in lymphoma (95). This strategy can be used to target stress-induced ligands like MICA and MICB on tumor cells (40, 96). Monoclonal antibodies against MICA and MICB can enhance the activation of NK cells and γδT cells, leading to the cytotoxicity of the tumor cells (97–99). To date, CLN619, the anti-MICA/B antibody, has shown promise in preclinical models and early clinical trials (100). ULBP is another family of NKG2DLs that can be targeted. ULBP1, ULBP2, and ULBP3 are expressed on various tumor cells and can be recognized by NKG2D. Targeting ULBP with specific antibodies as 23ME-01473 is undergoing a clinical trial, too. γδT cells cultured with artificial antigen-presenting cells and IL-2 show long-term proliferation (101).

### 4.3 Improving γδT cell persistence and *in vivo* expansion

One of the key limitations of γδT cell-based therapies is their limited *in vivo* persistence following infusion. Improving γδT cell persistence is critical for maximizing the therapeutic potential of γδT cell-based treatments.

#### 4.3.1 Cytokine support

The administration of cytokines such as IL-2, IL-15, and IL-21 has shown promising results in enhancing γδT cell persistence in clinical trials. For example, IL-15 has been extensively studied due to its ability to promote memory formation and long-term survival of γδT cells (102). A clinical trial demonstrated that IL-15 administration significantly improved the survival of infused γδT cells in patients with cancer and boosted their anti-tumor activity (103). IL-2 and IL-21 have also been used in combination with γδT cells, leading to enhanced proliferation and effector functions, which are essential for sustained immune responses (104). The clinical success of these cytokines in boosting γδT cell persistence highlights their importance in ensuring long-term anti-tumor effects in the clinical setting.

#### 4.3.2 Genetic modifications

Genetic modifications to γδT cells, particularly the expression of IL-15 or other survival cytokines, have shown great promise in improving the persistence and effectiveness of these cells post-infusion. For instance, recent study demonstrated that IL-15-engineered γδT cells exhibited self-sustained proliferation *in vivo*, thereby reducing the need for exogenous cytokines (105). These modified cells showed increased cytotoxicity and survival rates in preclinical models of tumor-bearing mice. Additionally, CRISPR/Cas9-mediated genetic modifications of γδT cells to express anti-tumor cytokines have been explored in early-phase clinical trials, with some studies showing enhanced expansion and persistence of the modified cells after infusion into patients. These advances in genetic engineering ensure that γδT cells have a sustained presence in the body, enhancing their ability to target and eliminate tumor cells.

#### 4.3.3 Biomaterial-based delivery

The use of biomaterial-based delivery systems, such as scaffolds or hydrogels, to encapsulate γδT cells has emerged as a promising strategy to improve their persistence and survival. Research has demonstrated that encapsulating γδT cells within biomaterials can sustain the release of cytokines, protect the cells from degradation, and promote local cell proliferation in the tumor site (106). For example, hydrogel-based delivery systems were used to enhance the persistence of immune cells, including γδT cells, in solid tumors. These materials provided a favorable microenvironment for γδT cells, leading to prolonged survival and enhanced anti-tumor responses in murine models (66, 107). Clinical research is ongoing, exploring biomaterial-based approaches for improving γδT cell function and longevity in tumor treatment, particularly for solid tumors with challenging microenvironments.

### 4.4 Overcoming functional heterogeneity of γδT cells

Not all γδT cells exhibit tumoricidal properties, some subsets, particularly those producing IL-10, can have immunoregulatory functions that suppress anti-tumor immunity, thus limiting their therapeutic potential (108). This heterogeneity complicates the clinical application of γδT cells and necessitates strategies to selectively expand the most cytotoxic subsets while minimizing the presence of regulatory subsets that may hinder therapeutic efficacy.

#### 4.4.1 Single-cell transcriptomics and proteomics

A promising approach to overcoming functional heterogeneity is the use of single-cell transcriptomics and proteomics. These technologies allow for the identification of tumor-reactive γδT cell subsets by profiling the gene expression and protein markers of individual cells (109). By using these methods, researchers can identify γδT cell subsets with optimal tumor-targeting properties, enhancing the precision and effectiveness of therapies. A study utilized single-cell RNA sequencing to identify tumor-specific γδT cell subsets in melanoma patients. They found that certain subsets of Vγ9Vδ2 γδT cells were highly activated in the presence of tumor antigens and produced pro-inflammatory cytokines, leading to tumor regression (75). This approach is being incorporated into ongoing clinical trials aiming to selectively expand the most effective γδT cell subsets for adoptive cell therapies, providing a more personalized and targeted approach to immunotherapy.

#### 4.4.2 Selective expansion of cytotoxic subsets

Selective expansion of cytotoxic subsets is a key strategy to enhance the therapeutic efficacy of γδT cells. By promoting the expansion of pro-inflammatory γδT cells that produce cytokines such as IFN-γ and TNF-α, while suppressing the expansion of regulatory subsets, this strategy focuses on maximizing cytotoxic potential while minimizing immune suppression (81). Recent research has shown that the expansion of γδT cells with cytokines such as IL-15 and IL-12 leads to the preferential growth of cytotoxic γδT cells, which exhibit superior anti-tumor effects. For example, clinical trials involving IL-12-stimulated γδT cells have demonstrated enhanced tumor killing and improved survival outcomes in patients with hematologic malignancies (110). Moreover, targeting regulatory γδT cells that produce IL-10 through specific cytokine manipulation has been shown to prevent immune suppression. The combination of IL-12 and IL-15 during γδT cell expansion selectively promoted cytotoxicity while reducing the presence of IL-10-producing regulatory subsets, significantly improving the efficacy of γδT cell therapy in solid tumor models (6).

### 4.5 Enhancing tumor infiltration and overcoming the immunosuppressive TME

A significant barrier to the efficacy of γδT cell-based immunotherapy is the immunosuppressive nature of the TME. To overcome this challenge, multiple strategies have been explored, with preclinical and clinical evidence supporting their potential to enhance γδT cell infiltration and function in the TME.

#### 4.5.1 Immune checkpoint blockade

One of the most promising strategies to counteract tumor-induced immunosuppression is the combination of γδT cell therapy with immune checkpoint inhibitors (ICIs) targeting PD-1, PD-L1, or CTLA-4. Checkpoint molecules such as PD-1 are upregulated in exhausted γδT cells within tumors, leading to reduced cytotoxic activity. Preclinical models have demonstrated that blocking PD-1 or PD-L1 signaling restores γδT cell function, leading to enhanced tumor clearance. Recent clinical trials have provided encouraging evidence for immune checkpoint blockade in combination with γδT cell therapy. In one study, anti-PD-1 therapy significantly enhanced the anti-tumor activity of γδT cells in non-small cell lung cancer (NSCLC) patients, leading to prolonged survival (111). Additionally, PD-L1 inhibitors have been shown to enhance the persistence and function of γδT cells in melanoma and hepatocellular carcinoma (HCC) models (112). These findings suggest that checkpoint blockade therapy can effectively reinvigorate γδT cells, improving their function within the immunosuppressive TME.

#### 4.5.2 Local tumor irradiation

Low-dose radiation therapy has been shown to modulate the tumor microenvironment and improve γδT cell-mediated immunity. One key mechanism involves the upregulation of NKG2D ligands, which enhance the recognition of tumor cells by γδT cells. Clinical studies have demonstrated the potential benefits of combining radiotherapy with γδT cell therapy. In patients with head and neck squamous cell carcinoma low-dose radiation can enhance NKG2D ligand expression, leading to improved γδT cell-mediated tumor clearance (83). Additionally, It has been shown that radiation therapy increases the susceptibility of tumors to γδT cell killing by inducing DNA damage and stress responses, making them more vulnerable to immune attack (113). This combination strategy is currently being tested in clinical trials for glioblastoma and lung cancer, with promising early results suggesting enhanced γδT cell infiltration and improved patient outcomes.

### 4.6 Advances in γδT cell-based therapies

Recent advancements in γδT cell-based immunotherapy have focused on optimizing adoptive cell therapy (ACT), developing chimeric antigen receptor (CAR)-γδT cells, and exploring combination strategies to enhance therapeutic efficacy. These innovations aim to overcome current limitations, such as limited persistence, tumor infiltration barriers, and immune suppression, while leveraging γδT cells’ unique ability to recognize stress ligands and tumor-associated antigens (**Table 1**).

**Table 1:** Strategies for improving γδT cells in tumor immunotherapy.

Category	Methods	Key Points & Representative Strategies
*In Vitro* Expansion and Activation		
Phosphoantigen (pAg)-based Therapy	- Use of phosphoantigens (IPP, DMAPP) or their derivatives to activate γδT cells	- Zoledronic acid (ZOL), BrHPP, and 2M3B1PP promote phosphoantigen accumulation, activating Vγ9Vδ2 T cells and enhancing cytotoxicity
Cytokine Stimulation	- Use of specific cytokines to promote γδT cell expansion	- IL-15 enhances survival and expansion of γδT cells
		- IL-12 and IL-18 increase IFN-γ and TNF-α secretion
		- Vitamin C boosts proliferation and cytotoxicity
Artificial APCs (aAPCs)	- Use of artificial antigen-presenting cells expressing co-stimulatory molecules and antigens	- NKG2D signaling enhances γδT cell activation and cytotoxicity
		- Anti-MICA/MICB antibodies (e.g., CLN619) are in preclinical and clinical trials
Adoptive Cell Therapy (ACT)		
γδT Cell Adoptive Transfer	- Isolation and ex vivo expansion of γδT cells for reinfusion	- γδT cells expanded using IPP or ZOL can be reinfused into patients, showing promise in early-phase clinical trials, especially for hematological cancers
CAR-γδT Cells	- Genetic modification of γδT cells to express chimeric antigen receptors (CARs)	- CAR-γδT cells combine γδTCR and CAR specificity to target tumor antigens, providing dual functionality
High-Affinity TCRs	- Transfection of αβ T cells with γδTCRs	- High-affinity Vγ9Vδ2 TCRs enhance tumor recognition and cytotoxicity
*In Vivo* Activation and Targeting		
Agonistic mAbs	- Monoclonal antibodies (e.g., anti-BTN3A1/CD277) to activate γδT cells	- ICT01 (anti-BTN3A1 mAb) is in phase I/IIa clinical trials for activating Vδ2 T cells
Bispecific Antibodies	- Bispecific antibodies linking γδT cells with tumor cells	- HER2-Vγ9 bispecific antibodies trigger HER2-expressing tumor cell killing
		- γδTCR-CD3 bispecific molecules (GABs) redirect αβT cells to attack tumors
Other Strategies		
Metabolic & Epigenetic Modulation	- Modulate γδT cells through metabolic and epigenetic pathways	- Histone deacetylase inhibitors (e.g., valproic acid) and DNA demethylating agents (e.g., decitabine) enhance γδT cell cytotoxicity
Enhancing Tumor Infiltration	- Improve γδT cell infiltration into tumor tissues	- Low-dose gamma irradiation enhances γδT cell recruitment
		- Hyaluronan synthesis inhibitors promote γδT cell penetration into tumors
Targeting Tumor Microenvironment	- Reverse immunosuppression in the tumor microenvironment	- Checkpoint inhibitors (anti-PD-1, anti-PD-L1, anti-CTLA-4) enhance γδT cell function by overcoming immune suppression
Combination Therapies	- Combine γδT cell therapy with other treatments	- Valproic acid synergizes with ZOL to enhance cytotoxicity
		- PARP inhibitors increase NKG2DL expression, improving tumor cell killing by γδT cells
Combination Therapies	- Combine γδT cell therapy with other treatments	- Valproic acid synergizes with ZOL to enhance cytotoxicity
		- PARP inhibitors increase NKG2DL expression, improving tumor cell killing by γδT cells

#### 4.6.1 Adoptive cell therapy

##### 4.6.1.1 Autologous *vs*. allogeneic γδT cell transfer

**Figure 3:**
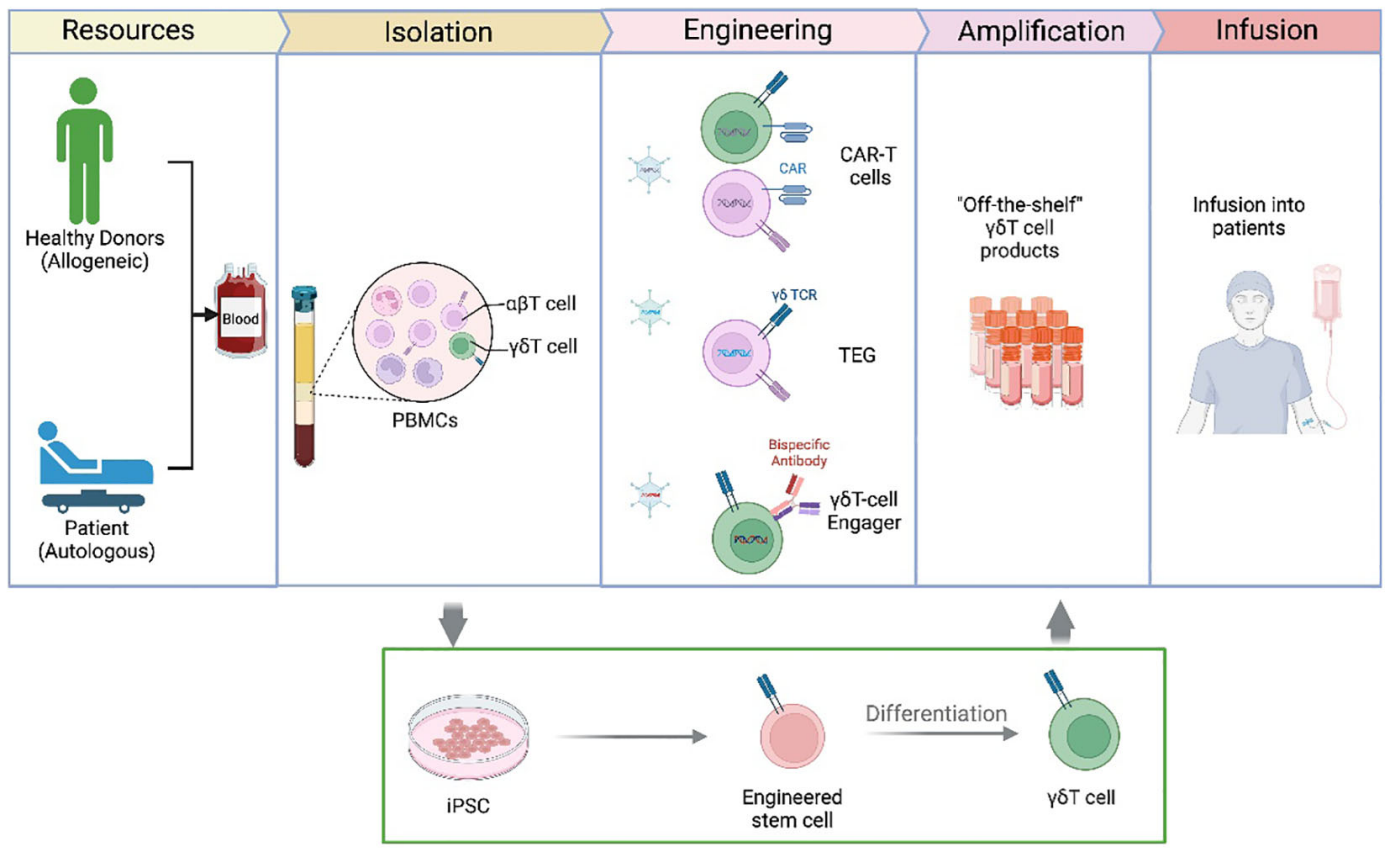
γδT Cell-based therapies and engineering approaches. This figure presents an overview of current γδ T cell-based immunotherapies, highlighting different strategies for their clinical application. γδ T cells can be isolated from peripheral blood mononuclear cells (PBMCs) from healthy donors (allogeneic) or patients (autologous). Following isolation, γδT cells can be engineered using: CAR-γδT cells – expressing chimeric antigen receptors to enhance tumor recognition; TCR-engineered γδT cells (TEG therapy) – modifying γδ TCRs for improved antigen specificity; or Bispecific antibody-based γδ T cell engagers – linking γδT cells to tumor cells via targeted antibodies. After ex vivo expansion and amplification, γδT cells are infused into patients as an “off-the-shelf” cellular therapy. Additionally, induced pluripotent stem cells (iPSCs) can be genetically engineered to generate γδT cells, offering an alternative approach for scalable production.

Traditional adoptive γδT cell therapy relies on autologous γδT cells, which are expanded from a patient’s own blood before reinfusion. However, autologous approaches are time-consuming and can yield inconsistent therapeutic responses due to variations in the patient’s immune status. To increase availability and streamline production, researchers are exploring allogeneic γδT cell therapy, using donor-derived γδT cells or off-the-shelf γδT cell products. Recent clinical trials have showed that allogeneic Vδ2 γδT cells expanded from healthy donors exhibited high cytotoxicity against hematologic cancers and had low risk of graft-versus-host disease (GVHD) due to the unique MHC-independent recognition mechanism of γδT cells (114). Additionally, induced pluripotent stem cells (iPSCs)-derived γδT cells are being developed as an off-the-shelf product, showing promising results in preclinical models of solid tumors and leukemia (77) (**Figure 3**).

##### 4.6.1.2 Introducing bispecific antibody

Bispecific antibodies represent an emerging approach to enhance γδT cell-mediated tumor targeting by bridging γδT cells with tumor cells. These antibodies are designed to bind both γδT cells and tumor antigens, directing γδT cells to tumor sites with greater specificity and cytotoxic efficiency. Bispecific Her2-Vγ9 antibody could trigger the killing of Her2-expressing tumor cells by Vγ9Vδ2 T cell lines (115, 116). Another study links the extracellular domains of tumor-reactive γ9δ2TCRs to a CD3-binding moiety, creating γδTCR anti-CD3 bispecific molecules (GABs) and it could redirect αβT cells against a broad range of tumors (117).

##### 4.6.1.3 Genetic engineering of γδTCRs: TCR-engineered γδT cells

Another approach to enhancing γδT cell specificity is TCR engineering, in which high-affinity γδTCRs are introduced into αβT cells to generate TEG cells. These engineered cells combine the tumor-targeting versatility of γδTCRs with the *in vivo* persistence and expansion capacity of αβT cells, resulting in a potent anti-tumor response (**Figure 3**). A study in acute myeloid leukemia (AML) reported that TEG cells targeting Wilms’ tumor antigen (WT1) successfully controlled leukemia progression in patients without severe toxicity (118).

#### 4.6.2 Combination therapies

##### 4.6.2.1 γδT cell therapy + checkpoint blockade

γδT cell express immune checkpoint inhibitors, including PD-1, particularly within the TME, where chronic antigen exposure can drive T cell exhaustion. However, unlike conventional αβT cells, γδT cells exhibit a distinct pattern of PD-1 expression and regulation, making their response to immune checkpoint inhibitors (ICIs) unique.

Recent studies have shown that PD-1^+^ γδT cells can display both exhausted and highly functional phenotypes, depending on co-stimulatory signals within the TME. While high PD-1 expression is often associated with reduced cytotoxicity, some tumor-reactive γδT cells retain effector function despite expressing PD-1. Checkpoint blockade using PD-1/PD-L1 inhibitors can reinvigorate γδT cell activity, restoring cytokine production and enhancing tumor killing (119).

A recent review highlighted that γδT cells engage with the PD-1/PD-L1 axis in a context-dependent manner, where PD-1 blockade can rescue γδT cell function in some tumors, while in others, additional co-stimulatory signals, like IL-15, NKG2D activation, may be required (119). Preclinical studies have demonstrated that blocking PD-1 in γδT cells enhances their cytotoxicity against ovarian cancer (120). Checkpoint blockade could also increase the expression of activating ligands on tumor cells, such as NKG2D ligands, making tumors more susceptible to γδT cell-mediated cytotoxicity (121–123).

##### 4.6.2.2 γδT cells + chemotherapy or radiation therapy

Combining γδT cell therapy with chemotherapy or radiation therapy is another strategy to enhance its effectiveness. Chemotherapy can cause tumor cell stress, leading to the upregulation of stress-induced ligands like MICA and MICB, which are recognized by γδT cells (124). This synergy can increase γδT cell activation and tumor cytotoxicity, potentially overcoming chemotherapy resistance mechanisms. Similarly, radiation therapy can increase tumor antigen release and the expression of immune-stimulatory molecules, making tumor cells more vulnerable to immune-mediated killing.

##### 4.6.2.3 γδT cells + metabolic modulation

The tumor microenvironment imposes metabolic constraints on γδT cells, including adenosine accumulation and TGF-β-mediated immune suppression, which inhibit γδT cell function (125). Targeting these pathways through metabolic modulation can enhance γδT cell survival and cytotoxicity.

Targeting adenosine A2A receptors (A2AR inhibitors) significantly boosted γδT cell cytotoxicity, leading to improved tumor regression in breast cancer models (126). Similarly, blocking TGF-β signaling was shown to prevent γδT cell exhaustion and enhance proliferation in pancreatic cancer models (107). These findings suggest that metabolic reprogramming could be a valuable adjunct to γδT cell immunotherapy, particularly for tumors with strong immunosuppressive microenvironments.

#### 4.6.3 Chimeric antigen receptor -γδT cells

Genetic modification of γδT cells to express CARs targeting tumor-specific antigens is an emerging strategy to enhance their tumor-targeting specificity and cytotoxic potential (73). While CAR-T cell therapy is for its application to αβT cells, CAR-γδ T can be designed to target specific tumor antigens, providing dual specificity through both the endogenous γδ TCR and the CAR (72). A recent preclinical study demonstrated that CAR-γδT cells targeting EGFRvIII in glioblastoma exhibited superior tumor eradication compared to conventional CAR-αβT cells (127). This highlights the potential advantages of CAR-γδT cells in solid tumors, where antigen escape is a common resistance mechanism. Clinical trials are now evaluating CD19- and BCMA-targeting CAR-γδT cells for B-cell malignancies, with early results showing promising tumor regression and minimal off-target toxicity.

## 5 Conclusion and future perspective

γδT cells represent a promising immune cell subset with unique tumor-targeting properties, particularly their ability to recognize cancer cells independently of MHC restriction. This characteristic allows them to overcome tumor heterogeneity and immune evasion, making them attractive candidates for immunotherapy. However, several challenges remain in fully translating γδT cell therapy into broad clinical application. One primary challenge is the variability of therapeutic effects across different tumor types, with some patients showing limited or poor responses to therapy (67). Additionally, the heterogeneity of γδT cells itself presents a complex challenge, as different subsets may exhibit distinct functional profiles, complicating their clinical use (128). Further research into the subtypes of γδT cells and their distinct roles in cancer immunity is essential to enhance efficacy across different cancer types.

A critical area of future research involves enhancing the persistence of γδT cells within the TME. Achieving sustained activity of γδT cells in TME is crucial for long-term therapeutic success, particularly in overcoming tumor recurrence and immune evasion (129). Strategies to optimize the trafficking, homing, and infiltration of γδT cells into solid tumors will be essential for improving clinical outcomes (130). Moreover, maintaining target antigen expression on tumor cells is key to preventing immune escape and ensuring durable responses.

Another critical area for advancement is minimizing the treatment -associated adverse effects, such as cytokine release syndrome (CRS) and other immune-related toxicities, which can arise from the activation and expansion of γδT cells (131). Understanding the mechanisms underlying these adverse reactions and developing strategies to mitigate them will be vital for improving the safety profile of γδT cell-based therapies.

The feasibility of non-viral gene transfer techniques for the generation of universal CAR γδT cells represents an exciting frontier. Non-viral methods could potentially overcome some of the limitations associated with viral vectors, such as immunogenicity and safety concerns, while enabling the development of off-the-shelf CAR γδT cell therapies. Advances in genome-editing technologies, such as CRISPR/Cas9, may play a pivotal role in this regard, facilitating the generation of more efficient and safer γδT cell therapies (132).

Despite these challenges, γδT cells offer a unique advantage in cancer treatment, particularly due to their ability to recognize and kill tumor cells without MHC restriction (108). This characteristic minimizes the risk of immune escape and addresses the issue of tumor heterogeneity. Meanwhile, ongoing clinical trials are assessing the safety and efficacy of γδT cell adoptive transfer in cancer patients, with early-phase studies demonstrating promising results, especially in combination with other immunotherapies.

In conclusion, the future of γδT cell-based cancer therapies holds great promise, with ongoing research aimed at optimizing their persistence, minimizing adverse effects, and exploring non-viral gene transfer techniques. By overcoming these hurdles, γδT cells could emerge as a transformative therapeutic approach for a wide range of cancers, offering new hope to patients who currently have limited treatment options.
